# Regulation of Thermal Emission Position in Biased Graphene

**DOI:** 10.3390/nano12193457

**Published:** 2022-10-03

**Authors:** Yansong Fan, Zhengzhuo Zhang, Zhihong Zhu, Jianfa Zhang, Wei Xu, Fan Wu, Xiaodong Yuan, Chucai Guo, Shiqiao Qin

**Affiliations:** College of Advanced Interdisciplinary Studies & Hunan Provincial Key Laboratory of Novel-Optoelectronic Information Materials and Devices, National University of Defense Technology, Changsha 410073, China

**Keywords:** graphene, thermal emission, regulation

## Abstract

A very attractive advantage of graphene is that its Fermi level can be regulated by electrostatic bias doping. It is of great significance to investigate and control the spatial location of graphene emission for graphene thermal emitters, in addition to tuning the emission intensity and emission spectrum. Here, we present a detailed theoretical model to describe the graphene emission characteristics versus gate voltages. The experimentally observed movement of the emission spot and temperature distribution of graphene emitters are basically in agreement with those from the theoretical model. Our results provide a simple method to predict the behavior of graphene emitters that is beneficial for achieving the spatial dynamic regulation of graphene infrared emission arrays.

## 1. Introduction

As a high-profile two-dimensional material, graphene has attracted extensive interest in worldwide for its excellent electrical, thermal, and optical properties [1,2,3,4]. In particular, controlling the Fermi level through electrostatic bias doping enables graphene to have a wide range of applications in electrically controlled devices [5].

In recent years, graphene thermal emission due to Joule heating has attracted a great deal of attention, and many studies have focused on enhancing the emission intensity or tuning the emission spectrum of heated graphene [6,7,8,9,10,11]. For example, a suspended structure in vacuum [6] or confined narrow bows structure [7,8] could sharply raise the electron temperature of graphene and make graphene emit bright light. Coupling graphene with many resonant structures, such as a Febry–Pérot cavity [9], photonic cavity [10], or metamaterial surface [11], could also modify graphene emission spectra from visible to mid-infrared. Moreover, the modulation speed of graphene-based on-silicon-chip blackbody emitters could reach 10 GHz at telecommunication wavelength [12].Meanwhile, it is very meaningful to investigate and dynamically control the spatial location of graphene emission for graphene thermal emitters [13]. The regulation of the thermal emission position in graphene could be used to realize novel electro-optical devices. Moreover, building a detailed theoretical model is helpful to understand the physics of graphene emission and quickly obtain the emission characteristics of graphene emitters.

In this work, we start with the charge conservation and tunability of carrier density and present a detailed theoretical model to calculate the distribution of the voltage, carrier density, locally generated heating power, and temperature along graphene. We find that this model can provide a clear and simple picture of the device behavior, in agreement with the results from simulations and experiments. For example, the calculated positions of minimized carrier density are consistent with those extracted from graphene infrared emission images. Under low gate voltages, maximum temperatures in the experiments are lower than those calculated by the temperature equation, but measured temperatures under high gate voltages are similar to those calculated by the equation. Moreover, the occurrence of another hot spot could be explained by simply introducing a locally trapped charges function into the carrier density equation. These results are helpful for realizing the controllable spatial emission of graphene emitters in the future.

## 2. Materials and Methods

**Figure 1:**
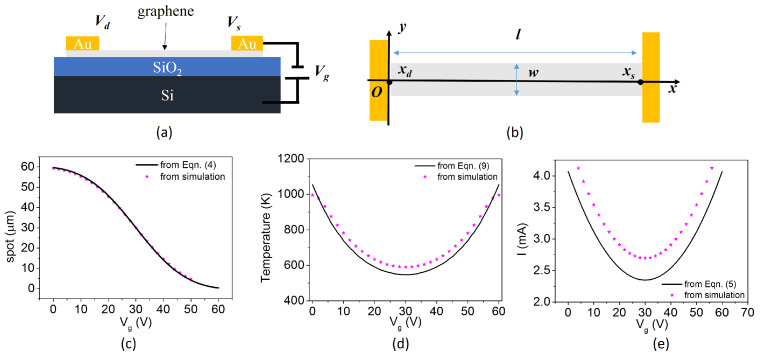
(**a**) A schematic of the graphene emitter. The electrical contacts are indicated as source, drain, and gate. (**b**) The top view of the graphene emitter; the comparison of from theoretical model and simulations for (**c**) spot position, (**d**) the maximum temperature, and (**e**) the current.

The theoretical model is based on the assumption that the carrier density of graphene is minimized when the Fermi level crosses the Dirac point, where the conduction band and valence band touch each other. For graphene thermal emitters on a $SiO_{2}$/Si substrate, as shown in Figure 1a, when bias voltage is applied to graphene, the current could be expressed as

(1) $$I=-\mu ew\cdot \eta \left(x\right)\frac{dV(x)}{dx}$$

where **x** is the direction along graphene (from drain to source), μ is the carrier mobility, η(x) is the carrier density, **V(x)** is the electrostatic potential, **e** is the electron charge, and **w** is the width of graphene. According to the charge conservation, the current is constant everywhere along graphene, so the carrier density depends on the electrostatic potential distribution, which could be written as [14]

(2) $$V\left(x\right)=V_{g}-V_{Dirac}+C_{ox}^{-1}\rho \left(x\right)e$$

where $V_{g}$ is the gate voltage, $C_{\mathrm{ox}}$ is the graphene capacitance, ρ(x) is the local carrier density, and $V_{\mathrm{Dirac}}$ is the Dirac voltage, which depends on the intrinsic doping level in graphene. The carrier density η(x) and local carrier density ρ(x) have the relationship of η(x)=±ρ(x), where + refers to the holes region and − refers to the electrons region.

Here, we ignore the recombination length of electrons and holes in graphene, so carriers are either electrons or holes but cannot be both. Therefore, the point that separates electron and hole regions has the lowest charge density, resulting in the highest resistance. According to charge conservation in the steady state and Joule heating, the current is equal everywhere and heating is proportional to the resistance, so the hottest place would occur at the point separating electron and hole regions. Meanwhile, according to Equation (2), only in the occasion of $V_{d}$ < $V_{g}$ – $V_{Dirac}$ < $V_{s}$ may the position separating electrons and holes be located on the graphene channel. It should be noted that some aspects of the real system have been ignored, such as contact resistance, the change of carrier mobility with position, and temperature.

Combining Equations (1) and (2) with boundary conditions, we can obtain the electrostatic potential expression along graphene and the position expression with minimum carrier density.

(3) $$V\left(x\right)=V_{g}-V_{Dirac}-\mathrm{sgn}\left(x-x_{0}\right)(\frac{2I}{\mu wC_{ox}}|x-x_{0}{\left|\right)}^{\frac{1}{2}}$$

(4) $$x_{0}-x_{d}=\frac{{\left(V\left(x_{d}\right)-V_{g}\right)}^{2}l}{{\left(V\left(x_{d}\right)-V_{g}\right)}^{2}+{\left(V\left(x_{s}\right)-V_{g}\right)}^{2}}$$

where **sgn(x)** is sign function, $x_{0}$ is the place of the minimum carrier density, $l=x_{s}-x_{d}$ is the length of graphene, and $x_{s}$ and $x_{d}$ are the edges of graphene and contacts.

The expressions for current, local carrier density, and Fermi level can also be derived as follows:

(5) $$I=\frac{w\mu C_{ox}}{2l}\left[{\left(V\left(x_{s}\right)-\left(V_{g}-V_{Dirac}\right)\right)}^{2}+{\left(V\left(x_{d}\right)-\left(V_{g}-V_{Dirac}\right)\right)}^{2}\right]$$

(6) $$\rho \left(x\right)=-sgn\left(x-x_{0}\right)\frac{1}{e}{\left(\frac{2C_{ox}I}{\mu w}\right)}^{\frac{1}{2}}|x-x_{0}{|}^{\frac{1}{2}}$$

(7) $$E_{f}\left(x\right)=sgn\left(x-x_{0}\right)\frac{\hbar v_{F}}{e}\sqrt{\pi |\rho (x)|}$$

Because the infrared emission intensity is related to the local Joule heating of graphene, and considering the presence of the carrier density ($n_{\mathrm{pd}}$) due to electron–hole puddles in graphene, the expression for locally generated power p(x) could be written as

(8) $$p\left(x\right)=IdV\left(x\right)/dx=\frac{I^{2}}{\mu ew\cdot \mathrm{sgn}\left(x-x_{0}\right)\cdot \sqrt{\rho {\left(x\right)}^{2}+n_{pd}^{2}}}$$

For graphene emitters on $SiO_{2}$/Si, most heat transfers into substrates, so the local temperature is

(9) $$T\left(x\right)=T_{sub}+\int \frac{p(x)w}{gh}dx$$

where $T_{\mathrm{sub}}$ is the substrate temperature, **g** is the effective thermal conductivity of the substrate, and **h** is the effective thermal conductance length.

So far, we have established the detailed theoretical model that contains a series of equations about the spot position, voltage potential, carrier density, Fermi level, locally generated power, and temperature distribution along the graphene emitter. For the case of $V_{g}$ – $V_{Dirac}$> $V_{d}$ > $V_{s}$ or $V_{d}$ > $V_{s}$ > $V_{g}$ – $V_{Dirac}$, according to Equation (2), we can conclude that the position of the minimum carrier density would always be close to the source or drain, which means the brightest spot would be always located there.

To verify the validity of the theoretical model, we also calculated the temperature distribution of the biased graphene emitter using finite-element simulation software (Comsol Multiphysics). In the simulation, different kinds of carrier density in graphene are taken into account with the following equations: [5,15].

(10) $$\begin{array}{c} J=\sigma E=ne\mu (n,T_{e})E\\ n=n_{e}+n_{h}\\ n_{e}\left(n_{h}\right)=\pm n_{d}+\sqrt{{n_{d}}^{2}+4{n_{th}}^{2}+{n_{pd}}^{2}}\\ n_{th}=\frac{\pi }{6}{\left(\frac{k_{B}T_{e}}{\hbar v_{F}}\right)}^{2}\left(1+e^{-(T_{e}/T_{0}-1)/2}\sqrt{T_{e}/T_{0}-1}\right)\\ V\left(x\right)=V_{g}-V_{Dirac}+C_{ox}^{-1}n_{d}\left(x\right)e\\ \mu \left(n,T_{e}\right)=\mu_{0}\times \frac{1}{1+{\left(\frac{n}{n_{ref}}\right)}^{\alpha }}\cdot \frac{1}{1+{\left(\frac{T_{e}}{T_{0}}-1\right)}^{\beta }} \end{array}$$

where σ is the conductivity of graphene, $n_{e}\left(n_{p}\right)$ is the electron (hole) density, $\mu (n,T_{e})$ is carrier mobility, $n_{\mathrm{th}}$ is the thermally excited carrier density of monolayer graphene, $n_{d}$ is the substrate doping carrier density, $T_{0}$ is the ambient temperature, $v_{F}$ is the Fermi velocity of graphene, and $\mu_{0}$ is the carrier mobility of graphene at 300 K. In the case of monolayer graphene on the $SiO_{2}$/Si substrate, the parameters are chosen as follows: $V_{Dirac}=-5$ V, $n_{pd}\approx 2.63\times 10^{11}$ cm${}^{-2}$, $\mu_{0}=1000$ cm${}^{2}$V${}^{-1}$s${}^{-1}$, $n_{ref}=1.1\times 10^{13}$ cm${}^{-2}$, *l* = 60 μm, w=5μm, $V_{d}$ = 70 V, $V_{s}=0$ V, $T_{0}=293$ K, α=2, beta=2.3.

The hot spot location, maximum temperature, and current calculated from equations and simulations are compared in Figure 1c–e. Except that the current from simulations is a little higher than that from Equation (5), the position and maximum temperature on graphene are almost identical to those obtained by equations, confirming the validity of our theoretical model.

## 3. Results

**Figure 2:**
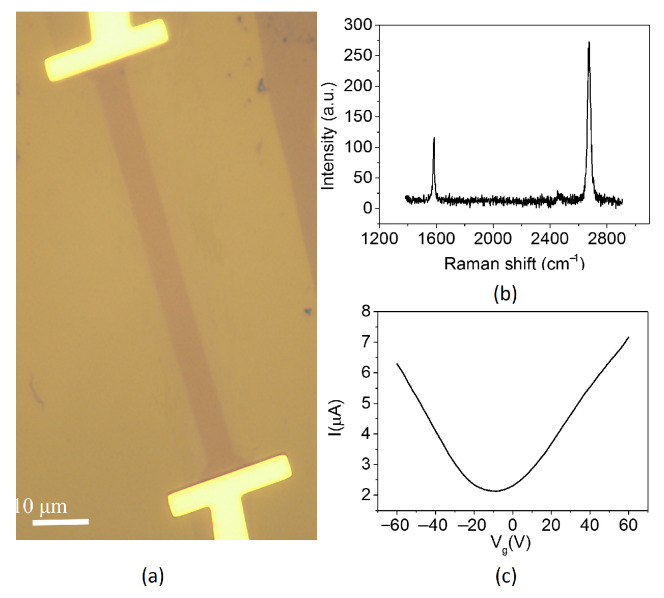
(**a**) The optical micrograph of the graphene thermal emitter with size of 60 μm × 5 μm; (**b**) Raman spectrum of the graphene emitter; (**c**) I-Vg characteristic with $V_{d}$ at 0.1 V after several annealing in vacuum.

A graphene flake was mechanically exfoliated on a p-doped Si substrate covered with a 285 nm thick $SiO_{2}$ layer, and metal contacts were defined by electron-beam evaporation of Cr (5 nm) and Au (50 nm). In order to avoid the overheating at contacts due to contact resistance, the graphene was etched to a shape narrow in the middle but wide on both sides, as shown in Figure 2a–c, which shows the Raman spectrum of the graphene emitter after fabrications and the field effect characteristic of the graphene emitter with a bias voltage at 0.1 V after annealing in a vacuum chamber.

**Figure 3:**
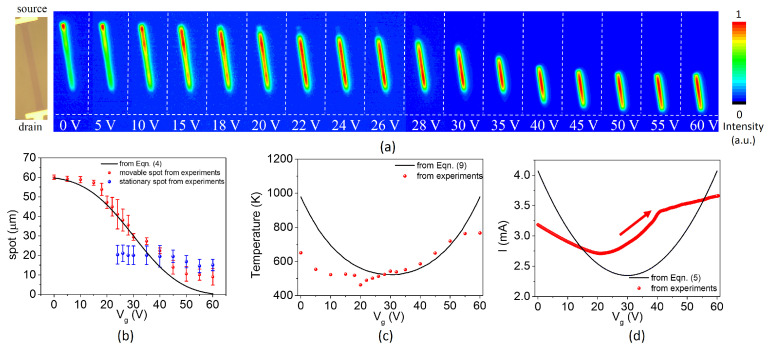
(**a**) Spatial images of the integrated infrared emission (with wavelength in the range from 950 nm to 1700 nm) of the graphene emitter with Vd of 70 V at different gate voltages; (**b**) the relationship between emission spots and the gate voltage; (**c**) the temperatures of movable spots and (**d**) the current dependent on the gate voltage.

A constant drain voltage of 70 V was applied to the graphene thermal emitter, while recording the spatial locations of graphene infrared emission as a function of applied gate voltages by an infrared CCD. All measurements were performed in vacuum to avoid the oxidation of graphene, and the emission intensity at each gate voltage in Figure 3a was normalized by the maximum intensity at the gate voltage. As shown in Figure 3a, a broad emission maximum appears close to the source contact for a gate voltage near 0 V, which moves along the channel for higher gate voltages and is close to the source when $V_{g}$ reaches 60 V. In order to clearly observe the movements of the emission spot, the positions of brightest spots in Figure 3a are extracted and plotted in red dots in Figure 3b, and the error bar represents regions where the emission intensity exceeds 95% of the maximum at the gate voltage. In addition to the movement from source to drain, the moving speed of the hot spot varies with the gate voltage: it is small when the hot spot is near the source or drain, while it is large when the hot spot moves to the middle of graphene.

Through correcting the infrared CCD with a blackbody (590 K–740 K), temperatures of hot spots in Figure 3) could be obtained and plotted with dots in Figure 3b. Although some measured temperatures at low gate voltages are lower than those from Equation (9), measured temperatures at higher gate voltages are close to those from Equation (9). The temperature deviation at low gate voltages could be attributed to the emission process from electron traps in the underlying $SiO_{2}$ substrate [16,17], as the experimental transfer current curve shifts by about 10 V compared with the result from Equation (5). Low current leads to a decrease of the maximum temperature of graphene.

**Figure 4:**
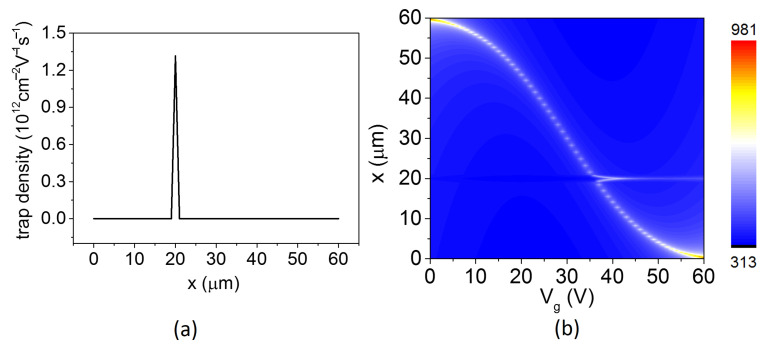
(**a**) The trapped charges as a function of position; (**b**) the temperature distribution with respect to the $V_{g}$ and position.

Interestingly, we found that a stationary light spot appeared at a place about 20 μm from the drain when extracting the brightest light from Figure 3a, as plotted with blue dots in Figure 3a. This spot almost remained unmoved when $V_{g}$ changed, which could be caused by locally trapped charges in the oxide that produce image charges in graphene [13]. In view of this, the density carrier equation could be modified by introducing a locally trapped charges function ($n_{trap}\left(x\right)$), as shown in Figure 4a. Then, Equation (6) is rewritten as

(11) $$\rho \left(x\right)=-sgn\left(x-x_{0}\right)\frac{1}{e}{\left(\frac{2C_{ox}I}{\mu w}\right)}^{\frac{1}{2}}|x-x_{0}{|}^{\frac{1}{2}}+n_{trap}\left(x\right)$$

The relationship between temperature and gate voltage is calculated using Equations (8), (9) and (11) and plotted in Figure 4b. As we can see, when $V_{g}$ is beyond 35 V, there are two hot spots on the graphene channel: one could move by increasing the gate voltage, and the other is stuck at the location with trapped positive charges. In fact, it is not enough to describe locally trapped charges by a fixed trapped charges function, because the locally trapped charges in the oxide could be influenced by many factors. Furthermore, the type of locally trapped charges in the oxide is likely to be converted from electron to hole as the $V_{g}$ changes, so the stationary spot could appear when $V_{g}$ is lower than 35 V. Regardless, introducing a locally trapped charges function into the density carrier equation is also meaningful to explain the stationary spot at high gate voltages.

## 4. Conclusions

In summary, we theoretically and experimentally investigated the spatial location of thermal emission in gate-controlled long channel graphene devices. The observed movement of the emission spot and temperature distribution are consistent with those obtained by the theoretical model, confirming that controlling the gate voltage is an effective method to tune the emission location and emission intensity. In addition, the relationship between the carrier density and Fermi level could be deduced by the theoretical model, which is helpful to understand the internal mechanism of graphene emission.

## Data Availability

Data supporting reported results can be found in Supplementary Materials.

## Supplementary Materials

The following supporting information can be downloaded at: https://www.mdpi.com/article/10.3390/nano12193457/s1, the detailed derivations and explanation of Equations (1)–(9) of the body text.
