# Supplementary material for: Regulation of Thermal Emission Position in Biased Graphene
Source: Nanomaterials (Basel). 2022 Oct 3;12(19):3457. doi: 10.3390/nano12193457 (PMC9565320; doi:10.3390/nano12193457)
Supplement: Supplementary file 1 [file nanomaterials-12-03457-s001.zip › nanomaterials-1951601-supplementary.pdf]

nanomaterials

MDPI

Supplementary materials

Regulation of Thermal Emission Position in Biased Graphene

Yansong Fan †, Zhengzhuo Zhang †, Zhihong Zhu, Jianfa Zhang, Wei Xu, Fan Wu, Xiaodong Yuan, Chucai Guo * and Shiqiao Qin

College of Advanced Interdisciplinary Studies & Hunan Provincial Key Laboratory of Novel-Optoelectronic Information Materials and Devices, National University of Defense Technology, Changsha 410073, China
* Correspondence: gcc_1981@163.com; Tel.: +86-731-8700-4211
† These authors contributed equally to this work.

In supplementary, we will show the detailed derivations of nine equations of the body text.

For a bias graphene on a SiO_2/Si substrate, the current density (***J***) be written as the product of the electrical conductivity (***σ(x)***) and the electric field (***dV(x)/dx***), i.e.

$$J = -\sigma(x)\frac{dV(x)}{dx} \quad \text{(S1)}$$

where ***x*** is the direction along graphene (from drain to source).

According to the micro-explanation of the electrical conductivity, ***σ(x)*** could be written as

$$\sigma = \mu e \cdot \eta(x) \quad \text{(S2)}$$

where ***μ*** is the carrier mobility, ***η(x)*** is the carrier density.

Considering a bias graphene whose length and width are ***l*** and ***w***, respectively, the current in graphene is

$$I = Jw = -\mu ew \cdot \eta(x)\frac{dV(x)}{dx} \quad \text{(S3)}$$

When large source-drain bias is applied to graphene, there would be a significant potential drop along the graphene, which could lead to the change of local charge density ***ρ(x)*** along graphene. So the voltage potential is a function of the local charge density and the gate voltage V_g, and could be written as

$$V(x) = V_g - V_{Dirac} + C_{ox}^{-1}\rho(x)e \quad \text{(S4)}$$

where C_{ox} is the graphene capacitance, ***ρ(x)*** is the local carrier density, and V_{Dirac} is the Dirac voltage, which depends on the intrinsic doping level in graphene. The carrier density ***η(x)*** and the local carrier density ***ρ(x)*** has the relationship of $\rho(x) = \pm\eta(x)$, where **+** refers to the holes region and – refers to the electrons region. According to Joule heating, the hottest place would occur at the point of highest resistance, i.e., the place of lowest carrier density.

Therefore, the charge density at the region which separates electrons and holes regions is lowest, resulting in the highest resistance at the region. So, the hottest place would occur at the point separating electron and hole regions.

Because we ignore the recombination length of electrons and holes in graphene, so carriers are either electrons or holes but cannot be both. According to the Equation (4), in the occasion of $V_d > V_s > V_g - V_{Dirac}$, the carriers in graphene are always holes, and the position of lowest carrier density is close to the source; in the occasion of $V_g - V_{Dirac} > V_d > V_s$, the carriers are electrons, and the position of lowest carrier density is close to the drain. In these two occasions, the position of lowest carrier density could not be changed by the gate voltage.

Therefore, only in the occasion of $V_d > V_g - V_{Dirac} > V_s$, there are both electron-conducting regions and graphene-conducting regions on the graphene, and the charge density of

the position separates electrons and holes regions is lowest. The location of lowest carrier density could be changed by the gate voltage. For the sake of convenience, we denote the position which separates electrons regions and holes as $\boldsymbol{x_0}$, and it is clear that the range of holes regions is $x_d < x < x_0$ and the range of electrons is $x_0 < x < x_s$.

So, for the holes regions, according to Eq (4), the carrier density could be written as

$$\eta(x) = \rho(x) = \frac{C_{ox}(V(x) - (V_g - V_{Dirac}))}{e} \quad \text{(S5).}$$

Bringing Eq (5) into Eq (3), we can obtain

$$I = -\mu w \cdot C_{ox}(V(x) - (V_g - V_{Dirac}))\frac{dV(x)}{dx} \quad \text{(S6).}$$

Integrating Eq (6), we can obtain

$$\frac{1}{2}[V(x) - (V_g - V_{Dirac})]^2 = -\frac{I}{mC}(x - x_0) \qquad x_d \le x \le x_0 \quad \text{(S7)}$$

and

$$V(x) = (V_g - V_{Dirac}) + (-\frac{2I}{\mu w C_{ox}}(x - x_0))^{\frac{1}{2}} \qquad x_d \le x \le x_0 \quad \text{(S8).}$$

Similarly, for the electrons regions, we can obtain

$$\eta(x) = -\rho(x) = -\frac{C_{ox}(V(x) - (V_g - V_{Dirac}))}{e} \quad \text{(S9)}$$

$$I = \mu w \cdot C_{ox}(V(x) - (V_g - V_{Dirac}))\frac{dV(x)}{dx} \quad \text{(S10)}$$

$$\frac{1}{2}[V(x) - (V_g - V_{Dirac})]^2 = \frac{I}{mC}(x - x_0) \qquad x_0 \le x \le x_s \quad \text{(S11)}$$

and

$$V(x) = (V_g - V_{Dirac}) - (\frac{2I}{\mu w C_{ox}}(x - x_0))^{\frac{1}{2}} \qquad x_0 \le x \le x_s \quad \text{(S12).}$$

Equations (8) and (12) could be reduced to an equation by introducing sign function.

$$V(x) = V_g - V_{Dirac} - \text{sgn}(x - x_0)(\frac{2I}{\mu w C_{ox}} | x - x_0 |)^{\frac{1}{2}} \quad \text{(S13)}$$

Because the voltage potential of edges ($\boldsymbol{x_d}$ and $\boldsymbol{x_s}$) of graphene and contacts also satisfies the equation (7) and (11), i.e.

$$\frac{1}{2}[V(x_d) - (V_g - V_{Dirac})]^2 = -\frac{I}{mC}(x_d - x_0) \quad \text{(S14)}$$

$$\frac{1}{2}[V(x_s) - (V_g - V_{Dirac})]^2 = \frac{I}{mC}(x_s - x_0) \quad \text{(S15).}$$

Combining the Eqs (14) and (15), position expression of the lowest carrier density could be obtained,

$$x_0 - x_d = \frac{(V(x_d) - V_g)^2 l}{(V(x_d) - V_g)^2 + (V(x_s) - V_g)^2} \quad \text{(S16)}$$

where $\boldsymbol{l = x_s - x_d}$ is the length of graphene.

Bringing Eq (17) into Eq (14) or (13), we can obtain current expression.

$$I = \frac{w\mu C_{ox}}{2l}[(V(x_s)-(V_g - V_{Dirac}))^2 + (V(x_d)-(V_g - V_{Dirac}))^2] \quad \text{(S17)}$$

According to Eq (4) and Eq (13), we can obtain the local charge density expression.

$$\rho(x) = -sgn(x-x_0)\frac{1}{e}(\frac{2C_{ox}I}{\mu w})^{\frac{1}{2}}|x-x_0|^{\frac{1}{2}} \quad \text{(S18)}$$

According to Eq (18), we can obtain the expression of Femi level of graphene.

$$E_f(x) = sgn(x-x_0)\frac{\hbar v_F}{e}\sqrt{\pi|\rho(x)|} \quad \text{(S19).}$$

Because the infrared emission intensity is related to the local Joule heating of graphene, and considering the presence of the carrier density (***n_{pd}***) due to electron-hole puddles in graphene, the expression for locally generated power ***p(x)*** could be written as

$$p(x) = IdV(x)/dx = \frac{I^2}{\mu ew \cdot \mathrm{sgn}(x-x_0)\cdot\sqrt{\rho(x)^2 + n_{pd}^2}} \quad \text{(S20).}$$

For graphene emitters on SiO_2/Si, most heat transfers into substrates, so the local temperature is

$$T(x) = T_{sub} + \int \frac{p(x)w}{gh}dx \quad \text{(S21).}$$

where ***T_{sub}*** is the substrate temperature, ***g*** is the effective thermal conductivity of the substrate and ***h*** is the effective thermal conductance length.

Until now, we have explained the derivations of equations (1) to (9) of the section of Materials and Methods of the body text.
